# Keep Moving Toward Recovery! Function-Focused Care in Hospitalized Stroke and Geriatric Patients

**DOI:** 10.1093/geroni/igaa057.2149

**Published:** 2020-12-16

**Authors:** Janneke de Man-van Ginkel, Carolien Verstraten, Marieke Schuurmans, Silke Metzelthin, Johannes Reitsma, Lisette Schoonhoven

**Affiliations:** 1 Julius Center for Health Sciences and Primary Care, UMC Utrecht, Utrecht, Utrecht, Netherlands; 2 University Medical Center Utrecht, Utrecht, Utrecht, Netherlands; 3 Maastricht University, Maastricht, Limburg, Netherlands

## Abstract

Many hospitalized patients experience decline in functional status. Function Focused Care (FFC) has demonstrated to improve patients’ functional status in long-term care. In a stepped wedge cluster trial in 893 hospitalized geriatric and stroke patients, we investigated the effectiveness of FFC compared to usual care (FFC: n=427, UC: n=466) on ADL and mobility. We measured the Barthel Index and the Elderly Mobility Scale, and analysed using a mixed-model multi-level method. At discharge, 3 month and 6 months, the mean difference (MD) was in favour of FFC, although at none of the time points the level of significance was reached: the MD for ADL was respectively: 0.79 (95%CI: -0.98-2.56), 0.43 (95%CI: 0.10-1.79), and 0.57 (95%CI: -1.34- 2.48). For mobility, the MD was respectively 0.89 (95%CI: -1.01-2.80), 0.78 (95%CI: -1.18; 2.75), and 1.09 (95%CI: -0.88-3.07). Although the results are inconclusive, FFC shows a tendency to improve ADL and mobility in hospitalized patients. Part of a symposium sponsored by Nursing Care of Older Adults Interest Group.

